# Commentary: PSD3 as a context-dependent modulator of immune landscape and tumor aggressiveness in esophageal squamous cell carcinoma

**DOI:** 10.3389/fimmu.2025.1687140

**Published:** 2025-10-07

**Authors:** Dongdong Zhang, Pei Zhang, Ran Jing, Ziwei Chen, Ming Cai

**Affiliations:** Department of Thyroid Oncology, Chongqing Key Laboratory of Translational Research for Cancer Metastasis and Individualized Treatment, Chongqing University Cancer Hospital, Chongqing, China

**Keywords:** esophageal squamous cell carcinoma, PSD3, PD-L1, TNFSF18, tumor microenvironment

## Introduction

The study by Luo et al. explores the dual role of PSD3 in esophageal squamous cell carcinoma (ESCC), positioning it as a context-dependent immuno-oncogenic factor that paradoxically correlates with improved survival while promoting tumor aggressiveness and suppressing PD-L1 (1). By integrating transcriptomic analyses (TCGA/GEO), functional assays, and spatial proteomics, the authors unveil a direct PSD3-PD-L1 interaction, suggesting novel therapeutic implications. This work advances ESCC immunobiology but faces methodological and translational challenges requiring scrutiny.

## Subsections relevant for the subject

First, The study leverages TCGA and GEO datasets to validate PSD3, CD274, and TNFSF18 expression in ESCC. While CD274 and TNFSF18 show consistent upregulation across cohorts, PSD3 exhibits discordance: significant overexpression in TCGA but no difference in GEO. The authors attribute this to “context-dependency” yet omit technical biases (e.g., batch effects in GEO microarray vs. TCGA RNA-seq) (2). Such variability undermines PSD3’s reliability as a universal biomarker. Future studies should harmonize platforms and include proteomic validation to resolve discrepancies.

Second, a key paradox emerges: PSD3 knockdown suppresses ESCC proliferation/invasion *in vitro*, yet high PSD3 correlates with longer overall survival. The authors speculate this may reflect PSD3’s immune-modulatory role (e.g., PD-L1 suppression) but lack *in vivo* evidence. Murine models (e.g., immunocompetent C57BL/6N mice) could clarify whether PSD3’s pro-survival effect stems from enhanced anti-tumor immunity. Without this, the clinical relevance remains speculative.

Third, immune deconvolution (ImmuCellAI/TIMER2.0) links PSD3 to immunosuppressive cells (Tregs, M0 macrophages) but shows no association with CD8+ T/NK cells (3). The authors note this may reflect algorithmic constraints but fail to validate with spatial methods (e.g., multiplex IHC for CD8+ T cells). Additionally, while PSD3 inversely regulates PD-L1, its impact on T-cell exhaustion markers (e.g., TIM-3, LAG-3) is unexamined. Single-cell RNA-seq could resolve cellular heterogeneity and clarify PSD3’s immune role.

Fourth, Co-IP confirms physical interaction between PSD3 and PD-L1, yet the regulatory mechanism remains unclear. Does PSD3 degrade PD-L1? Modulate its trafficking? The study neglects rescue experiments (e.g., PD-L1 overexpression in PSD3-knockdown cells). Furthermore, PSD3’s role in other immune checkpoints (e.g., CTLA-4) is ignored (4). Structural biology approaches could map binding interfaces to guide therapeutic disruption.

Finally, although PSD3 is proposed as a therapeutic target, no pharmacological modulators are tested. The lack of *in vivo* efficacy data (e.g., PSD3 inhibition in syngeneic tumors) limits clinical extrapolation. Additionally, cohort heterogeneity (e.g., HPV status, treatment history) may confound survival associations. Prospective validation in immunotherapy-treated ESCC cohorts is needed to assess PSD3’s predictive value for checkpoint blockade response (5).

## Discussion

This study innovatively positions PSD3 as a Janus-faced modulator of ESCC progression and immunity. Its strengths lie in multi-modal validation (bioinformatics, spatial proteomics, functional assays) and the discovery of the PSD3-PD-L1 axis. However, cohort discrepancies, unresolved survival paradoxes, and unaddressed mechanistic questions hinder translational potential. Future work should: 1). Employ spatially resolved techniques (e.g., CODEX) to map PSD3/PD-L1 dynamics in immune niches. 2). Validate findings in genetically engineered mouse models with lineage tracing. 3). Screen for PSD3 inhibitors and evaluate combinatorial efficacy with anti-PD-1 therapy. By addressing these gaps, PSD3 could emerge as a viable target for precision immuno-oncology in ESCC.

## References

[1] Luo S Li H Cai B Nurbahati A Cui H Peng T. PSD3 as a context-dependent modulator of immune landscape and tumor aggressiveness in esophageal squamous cell carcinoma. Front Immunol. (2025) 16:1641254. doi: 10.3389/fimmu.2025.1641254, PMID: 40895529 PMC12394057

[2] Sprang M Andrade-Navarro MA Fontaine JF. Batch effect detection and correction in RNA-seq data using machine-learning-based automated assessment of quality. BMC Bioinf. (2022) 23:279. doi: 10.1186/s12859-022-04775-y, PMID: 35836114 PMC9284682

[3] Li T Fu J Zeng Z. TIMER2.0 for analysis of tumor-infiltrating immune cells. Nucleic Acids Res. (2020) 48:W509–14. doi: 10.1093/nar/gkaa407, PMID: 32442275 PMC7319575

[4] Munari E Mariotti FR Quatrini L. PD-1/PD-L1 in cancer: pathophysiological, diagnostic and therapeutic aspects. Int J Mol Sci. (2021) 22:5123. doi: 10.3390/ijms22105123, PMID: 34066087 PMC8151504

[5] Zheng S He S Liang Y. NME4 suppresses NFκB2-CCL5 axis, restricting CD8+ T cell tumour infiltration in oesophageal squamous cell carcinoma. Immunology. (2024) 173:408–21. doi: 10.1111/imm.13838, PMID: 39016535

